# Influence of vertical wind shear on wind- and rainfall areas of tropical cyclones making landfall over South Korea

**DOI:** 10.1371/journal.pone.0209885

**Published:** 2019-01-07

**Authors:** Dasol Kim, Chang-Hoi Ho, Doo-Sun R. Park, Jinwon Kim

**Affiliations:** 1 School of Earth and Environmental Sciences, Seoul National University, Seoul, South Korea; 2 Department of Earth Sciences, Chosun University, Gwangju, South Korea; 3 National Institute of Meteorological Sciences, Korea Meteorological Administration, Jeju-do, South Korea; Peking University, CHINA

## Abstract

The wind- and rainfall areas of tropical cyclones (TCs) making landfall over South Korea were examined for the period 1998–2013 by using the Modern Era Retrospective Analysis for Research and Applications, version 2 (MERRA-2) and Tropical Rainfall Measuring Mission (TRMM) 3B42 data. Here, the wind- and rainfall areas were defined as the regions where wind speeds and precipitation rates exceed 14 m s^-1^ and 80 mm day^-1^ within 1000 km from the TC center, respectively. In general, TCs show significantly asymmetric wind and rainfall structures, with strong vertical wind shear appearing over South Korea during the landfall period. The rainfall area significantly increases with environmental vertical wind shear while the wind area is not sensitive to it. Composite analyses of the cases of strong and weak vertical wind shear confirm that the increase of rainfall area is related to the asymmetric convection (rising/sinking motion in the downshear-left/upshear-right side) induced by the vertical wind shear. This work highlights the importance of local atmospheric environment in determining the area primarily affected by strong winds or heavy rainfall during TC landfalls.

## Introduction

The extent of areas affected by a tropical cyclone (TC) at the time of its landfall is a critical factor in determining the damages caused by the TC [1–3]. Despite its importance, areal extents of TCs, however, have received much less attention than other TC properties such as tracks and intensities (e.g., maximum wind speed or minimum central pressure). Only a few meteorological agencies forecast TC size although many of them forecast TC tracks and intensities. Here, ‘size’ is a general terminology representing the areal extent of a TC, typically in terms of the radius of a threshold wind speed (e.g., a gale-force wind of 17 m s^−1^) or the radius of an outer closed isobar (ROCI) [4, 5]. For example, the National Hurricane Center of the United States, a leading agency in TC predictions, produced formal verifications of the wind radii forecasts only recently [6]. According to their results, statistical methods can skillfully predict wind radii of TCs over 72-hour span even if the performance of dynamical models is not satisfactory. However, the statistical methods largely rely on the predictability of the TC intensity, and consider only a few factors such as latitudes, and storm speeds that affect TC size [7].

To develop an accurate forecast system for the areal extents of TCs, it is necessary to understand how they vary according to environmental conditions. Earlier studies showed that the areal extent of a TC varies independently of the TC intensity [4, 5, 8–11]. Areal extents of TCs also vary following regions (e.g., ocean basins, latitudes), seasons, and the development stage [5, 9–13]. Recently, several studies explored the physical processes and mechanisms related to the variations of the areal extents of TCs [14–18]. Lin et al. [19] and Chavas et al. [11] suggested that local sea surface temperatures (SSTs) relative to the mean SST over the tropics are the primary factor controlling the areal extents of TCs in the tropics. Kim et al. [20] suggested the areal extents of TCs in the subtropics is significantly affected by environmental flow (i.e., steering flow and vertical wind shear) rather than by the SST, since TCs in the subtropics are exposed to significantly cooler SSTs and stronger environmental flow which induces more asymmetric TC structure compared to the tropics.

Despite recent studies, there is a major hurdle in studying the areal extents of TCs, due to limited amounts of observational data. Observation systems over land such as weather stations or radars can provide accurate high-resolution data in both time and space. However, they can observe a TC only when the TC is close to the stations or radars, thus have difficulties in covering the entire area of a TC. Aircraft observations and dropsonde are useful to investigate 3-dimensional structure of a TC, but they are also limited in sampling frequencies and locations. Satellite observations have made the most significant contributions to understanding on characteristics of the areal extents of TCs. For example, TC sizes in terms of the radius of a threshold wind speed can be obtained from satellite-retrieved near-surface winds [10, 11, 21, 22]. Chan and Chan [22] investigated the TC size (mean radii of 17 m s^-1^) using the Quick Scatterometer (QuikSCAT) data for the period 1999–2009 to suggest spatially and seasonally varying characteristics of TC sizes in global oceans. Chavas et al. [11] also examined outer sizes of TCs (radii of 12 m s^-1^ and vanishing wind) in a global perspective using the QuikSCAT data. However, satellite data also suffer from limitations as they cannot provide wind data over lands and can observe TCs twice a day at the most with limited spatial coverages [6, 23].

As an alternative to the limited observations, recent studies have suggested the possibility of using reanalysis data to investigate the areal extents of TCs. For example, the spatial and temporal distributions of TC positions in reanalysis data compare well with those in the best track data [24]. Significant correlation was also found between the TC intensity in reanalysis data and in the best track data although the TC intensities in reanalysis data are generally weaker than those in the best-track data [25]. The thermal and dynamical structures of TCs such as robust warm cores, primary and secondary circulations are also well presented in reanalysis data [26]. As the current reanalysis data can reliably resolve outer circulation of TCs, Knaff et al. [27] used the magnitude of the outer circulation of a TC—the 850-hPa mean tangential wind at a radius of 500 km—in reanalysis data as a proxy for TC size. In addition, Loridan et al. [28] investigated asymmetric wind structures of TCs based on reanalysis data.

Another issue regarding the areal extents of TCs is the rainfall area induced by the TCs. The areal extent of a TC has been typically defined in terms of TC wind structures such as the radius of a threshold wind speed and ROCI. However, the spatial distributions of TC winds and their variations are different from those of TC rainfall [29, 30]. For example, Ying et al. [31] found the opposite long-term changes in the annual accumulated wind and rainfall induced by TC landfalls in China. Similarly, Kim et al. [32] showed that TC-induced heavy rainfall in South Korea has significantly increased since 1970s, while the maximum wind speed has not. These studies raise the necessity of thorough investigation of the areal extend of rainfall and winds during TC landfalls. There exist only a limited number of studies on quantitative investigations on the TC rainfall area [19, 20, 30, 33, 34].

This study aims to investigate the areal extents of winds and rainfall induced by TCs making landfall over South Korea and to find possible explanations for their variations. South Korea is frequently affected by TCs, about 3 TCs a year to suffer considerable damages. For example, TCs RUSA in 2002 and MAEMI in 2003 caused economic losses of 41 and 35 billion USD, respectively. In addition, large baroclinic environments prevail over South Korea to significantly affect the characteristics of TCs (e.g., track, wind and rainfall intensity) in this region [32, 35, 36]. For example, Kim et al. [32] suggested the effects of an upper-tropospheric trough can significantly contribute to the variations of TC rainfall over South Korea. Park et al. [37] also showed that extratropical transition (ET) occurs frequently over South Korea, and that the spatial distributions of winds and rainfall are significantly different between TCs with and without ET process. These studies were mainly based on observational data from weather stations in South Korea and did not examine the details of the wind and rainfall structures. This study focuses specifically on the influence of baroclinic environment on wind and rainfall structures of landfalling TCs. This study utilized data from reanalysis and satellite retrievals for the wind- and rainfall areas of landfalling TCs due to the limitations in observed data.

## Materials and methods

### TC information

**Fig 1:**
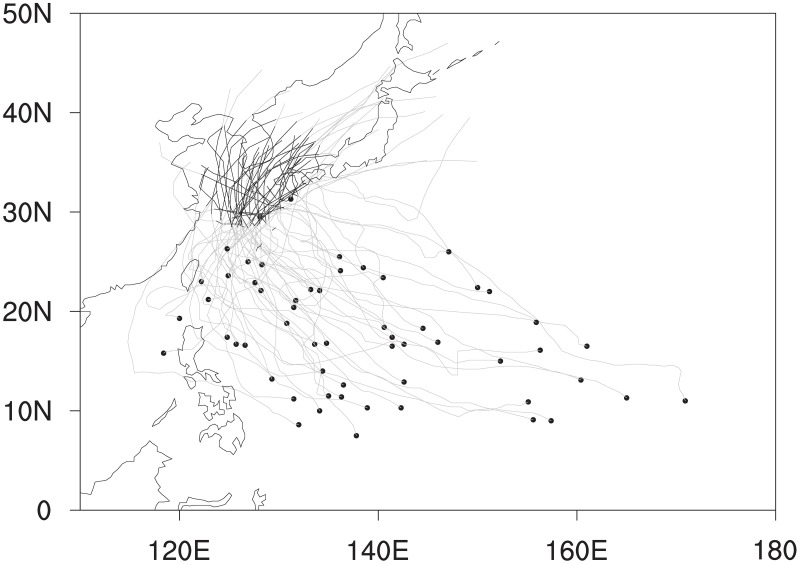
Tracks of TCs making landfall over South Korea during 1998–2013. The black and gray lines indicate tracks of landfall period and entire life time of each TC, respectively. Dots denote the genesis locations of TCs.

The best track data from the Regional Specialized Meteorological Center’s (RSMC) Tokyo Typhoon Center were used to identify TCs which made landfall over South Korea. This data provide information on individual TC such as the location of TC centers (in both latitude and longitude), the 10-min averaged maximum sustained wind speed, and the minimum sea level pressure (SLP) at 6-h intervals over the western North Pacific Ocean from 1951 to present. From this data, only TCs classified as the tropical storm, severe tropical storm, or typhoon are selected for investigation. In this study, the TC landfall over South Korea is defined as the period during which the distance between the TC center and the nearest coastline of South Korea is less than 500 km. This definition deviates from the strict definition of TC landfall (i.e., the timing when the TC center encounters a coastline), but is consistent with a number of studies in which similar definitions of TC landfall are used because the impact of a TC can occur even before the TC hits the coastline [2, 32, 37]. Fig 1 shows the tracks of 57 TCs selected based on this definition for the analysis period 1998–2013.

### Wind and rainfall data

**Fig 2:**
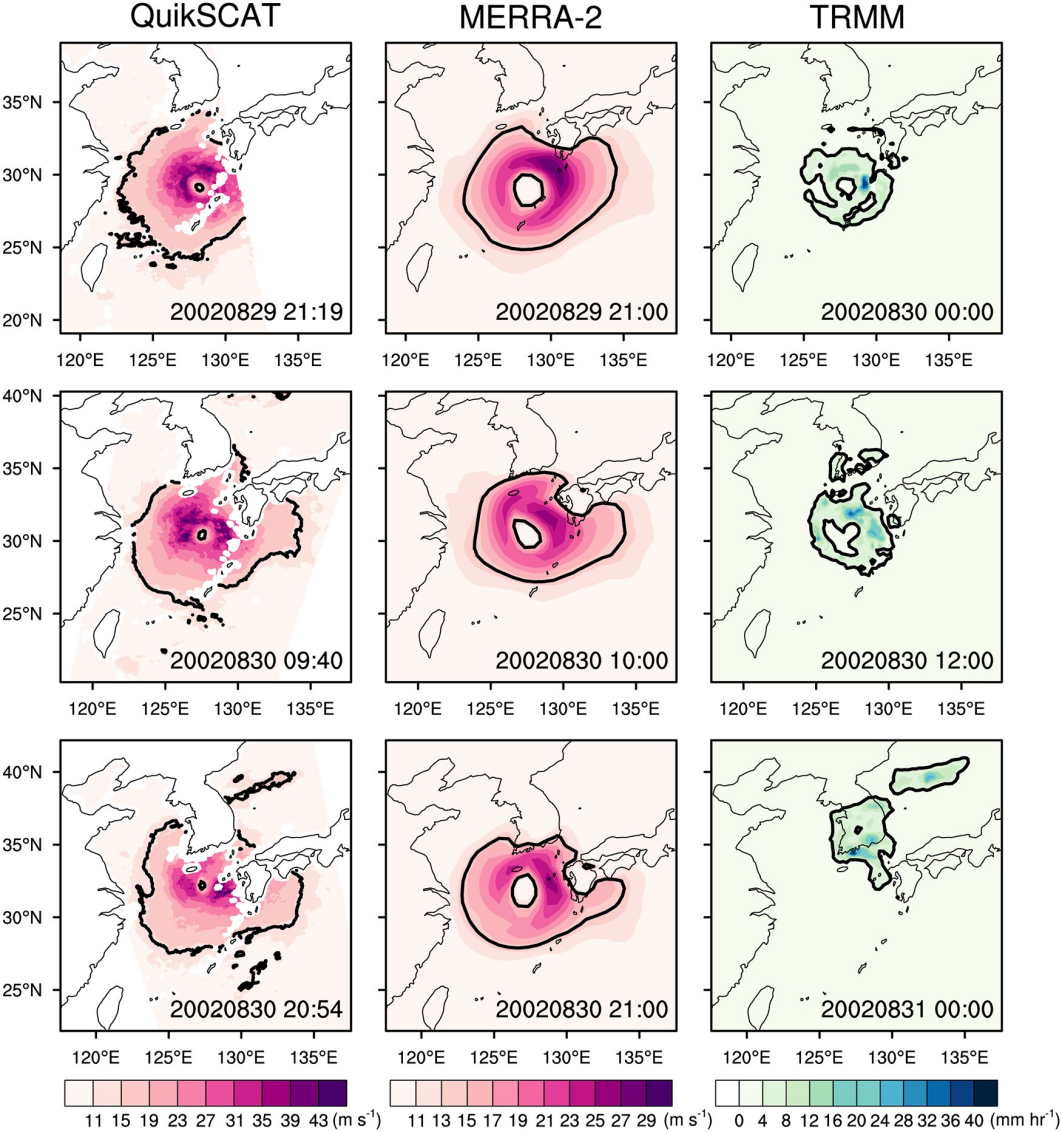
Spatial distribution of wind speed (unit: m s^-1^) at 10-m level, and precipitation rate (unit: mm hr^-1^) for the RUSA (2002) obtained from the QuikSCAT version 3 (left), MERRA-2 (middle), and TRMM-3B42 (right) data, respectively. The time of the MERRA-2 and TRMM-3B42 data is selected to be closest to that of the QuikSCAT data. Black contour lines indicate the region where wind speed and precipitation rate is 14 m s^-1^ and 3.33 mm hr^-1^, respectively.

The observational data for the wind- and rainfall areas affected by TC landfalls are severely limited, especially for wind structures. Observational network over the land usually have limited coverage of the area affected by a landfalling TC. The satellite scatterometers can estimate the surface winds, but only over the ocean. Thus, it is difficult to examine the 3-dimensional wind structure of TCs using these observational data. Because of these difficulties, this study utilizes the Modern Era Retrospective Analysis for Research and Applications, version 2 (MERRA-2) [38] reanalysis data from the Global Modeling and Assimilation Office (GMAO) to investigate the 3-dimensional wind structure of TCs and the large-scale atmospheric environments. The horizontal resolution of the MERRA-2 reanalysis data is 0.5° latitude × 0.625° longitude. To examine the dynamic structure of a TC, horizontal winds at 10-m, and 250 hPa level, vertical velocity at 500 hPa level were obtained from the MERRA-2 reanalysis data at 6-h intervals along the TC center in the RSMC best track data. Schenkel and Hart [25] reported differences in the TC center positions between the reanalysis and best track data; however this may not significantly affect our results since a sufficiently large area (i.e., the circle of radius 1000 km in Fig 2) around each TC center was taken into account when calculating the areal extents of the wind and rainfall associated with the TC. It is also noteworthy that the average difference of TC locations near South Korea is less than 75 km in the MERRA reanalysis according to Schenkel and Hart [25] (see their Fig 1e). The daily maximum wind data observed by the 69 weather stations in South Korea and QuikSCAT version 3 [39] produced by the Jet Propulsion Laboratory, National Aeronautics and Space Administration were also used to compare the wind structures of TCs in the MERRA-2 reanalysis data and those in the observation.

The rainfall data were obtained from the Tropical Rainfall Measuring Mission (TRMM) 3B42 which is 3-h interval precipitation data retrieved from TRMM and other multi-satellite [40]. The TRMM-3B42 has been used in many studies on the TC rainfall due to its high temporal resolution and wide spatial coverage [19, 33, 34, 41–43]. This data also can detect intense TC rainfall reasonably, although it shows higher skills over the ocean than the land [44, 45]. The temporal and geographical coverage of this data are from January 1998 to September 2014 and the global band from 50°S to 50°N, respectively. The TRMM data limit the analysis period to 1998–2013 and the latitudinal coverage to 50°N in this study. Note that the center of TC was limited to 40°N to allow the TC radius of 1000 km used in calculation of the areal extents of wind and rainfall (next section). The horizontal resolution of this data is 0.25° latitude × 0.25° longitude. The TRMM data are interpolated to the grid of MERRA-2 reanalysis to be consistent with the MERRA-2-based areal extent of wind calculations.

### Definition of areal extents of wind and rainfall

Fig 2 illustrates an example of the areal extents of wind and rainfall (i.e., black contour lines) in this study. The areal extent of wind (i.e., wind area) was defined as, within 1000 km from a TC center, the total size of regions in which wind speed exceeds 14 m s^−1^. Previous studies used various wind speed thresholds, for example, 17 m s^-1^ [10, 22], 15 m s^-1^ [13], 12 m s^-1^ [11, 21], 5 knots [27], to calculate the wind area. The larger threshold value is used to measure the inner region of a TC. The threshold used in this study is close to the values for defining outer areas of TCs (e.g., 15 and 12 m s^-1^) and corresponds to the high-wind of the Beaufort wind scale. The areal extent of rainfall (i.e., rainfall area) was similarly defined as, also within 1000km from a TC center, the total size of regions where rainfall exceeds 80 mm day^−1^ (= 3.33 mm hr^−1^), the threshold value of heavy-rainfall used in the Korea Meteorological Administration. This is analogous to the definition for the TC rainfall area used in Matyas (36, 37). The overall results are not much sensitive to the threshold values for defining the wind- and rainfall areas. Thresholds within the range of 12–15 m s^-1^ and 2–5 mm hr^-1^ yield essentially the similar results. The wind- and rainfall areas were calculated at every six hours for the periods in which TC landfalls occurred in South Korea. This resulted in 283 samples for the wind- and rainfall areas for analysis.

The use of the wind- and rainfall areas instead of more conventional definition of areal extent in terms of the mean radius of the threshold wind or rainfall, is for considering asymmetry of the wind and rainfall fields of TCs [46–51]. Asymmetry of a TC is represented by several wavenumber components, but the wavenumber-1 component can usually explain most of it [51, 52]. When the wind or rainfall field of a TC has wavenumber-1 asymmetry, the radial size may not be suitable for representing the real area of a TC. For example, the radius of threshold wind speed (R) with wavenumber-1 asymmetry can be simplified as the following equation [20]. $R=\overset{-}{R}(1+\alpha \text{sin}\left(\theta \right))$, where $\overset{-}{R}$ is the azimuthal mean radius of threshold wind speed, α is the magnitude of the wavenumber-1 asymmetry, and θ is the azimuth angle from the center of the TC. According to the conventional definition, the radial size of TC is $\overset{-}{R}$, and the corresponding area is $\pi {\overset{-}{R}}^{2}$, but the real area surrounded by the R is $\pi {\overset{-}{R}}^{2}(1+\frac{1}{2}\alpha^{2})$. When α = 1, the areal extent can be 1.5 times larger than that estimated without considering the asymmetry. This distortion of the areal extent by using the radial size can occur in real TCs even though the real structure of wind or rainfall is more complicated and the magnitude of asymmetry is smaller than this simple case [20, 51, 52].

## Results

### Comparison of TCs in MERRA-2 reanalysis and observation

Characteristics of TCs in MERRA-2 show a robust relationship with those in the observation during the period of landfalls in South Korea. For example, the TC intensity of the MERRA-2 presents significant correlation with that of the best track data at the 95% confidence level (correlation coefficient, *r* = 0.632 and 0.627 for maximum wind speed and minimum central SLP, respectively). Here, the TC intensity of the MERRA-2 was calculated as the 10-m maximum wind speed and minimum central SLP within a radius of 300 km from the TC center. The daily maximum wind speeds over South Korea obtained from the 69 weather stations during TC landfall are also significantly correlated (*r* = 0.67) with those from the MERRA-2, although the absolute values in MERRA-2 are smaller (linear regression slope = 0.49) than the observed (not shown). In case multiple weather stations are located in the same MERRA-2 grid box, the daily maximum wind for weather stations was calculated as the average of all stations within the grid box.

Fig 2 shows the horizontal distribution of the 10-m wind speeds and precipitation rates for August 29–30, 2002, associated with the RUSA (2002), from the QuikSCAT, MERRA-2, and TRMM-3B42 data. Note that the QuikSCAT can retrieve winds only over ocean surfaces, and thus cover only partial areas of the TC during the landfall (Fig 2a, 2d and 2g). Since the swath of the QuikSCAT is 1800 km [53], the QuikSCAT may not cover the entire TC if the satellite passed far from the TC center (Fig 2a). Thus, it is advantageous to use reanalysis data which are uniform in time and space in analyzing the areal extent of a TC. The wind speed, especially the maximum wind speed, in MERRA-2 are generally weaker than those in the satellite data mainly due to the limited horizontal resolution of the reanalysis data [25, 26] and the fact that the reanalysis data represent the average values within the grid box. However, both reanalysis and satellite data yield similar wind patterns, especially for their areal extents (black contour line). This result denotes the MERRA-2 can resolve outer circulation of TC reasonably. The reliability of reanalysis data in resolving outer circulation of TC also can be found in previous studies [27, 54].

### General characteristics of wind- and rainfall areas

**Fig 3:**
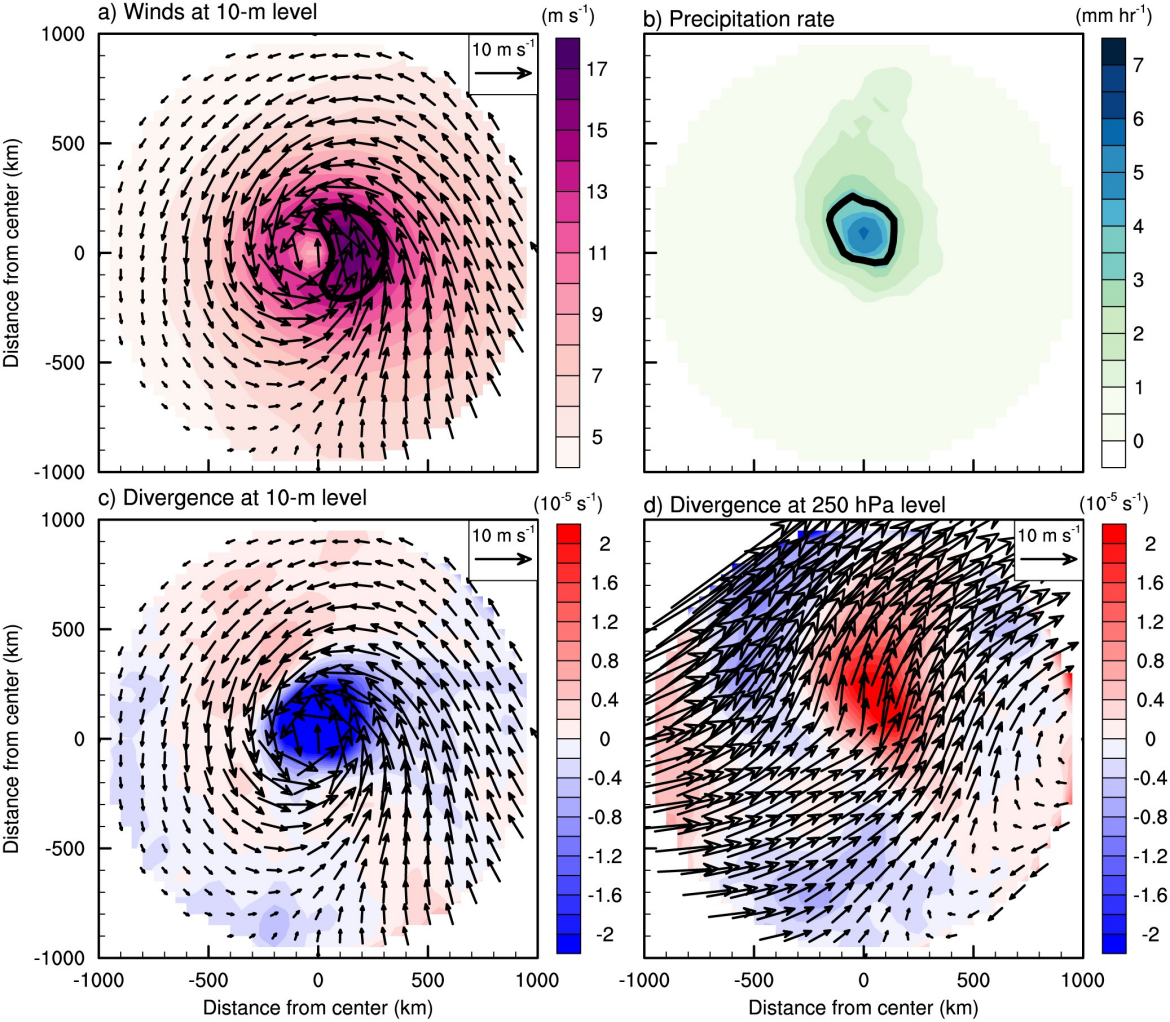
The averaged horizontal TC structures of (a) wind at 10-m level (arrows, unit: m s^-1^) with its speed (shaded, unit: m s^-1^), (b) precipitation rate (unit: mm hr^-1^), and divergence (shaded, unit: 10^−5^ s^-1^) and wind (arrows, unit: m s^-1^) at (c) 10-m and (d) 250 hPa level during the landfall period. The directions of x- and y- axis are right and front to TC motion, respectively.

**Fig 4:**
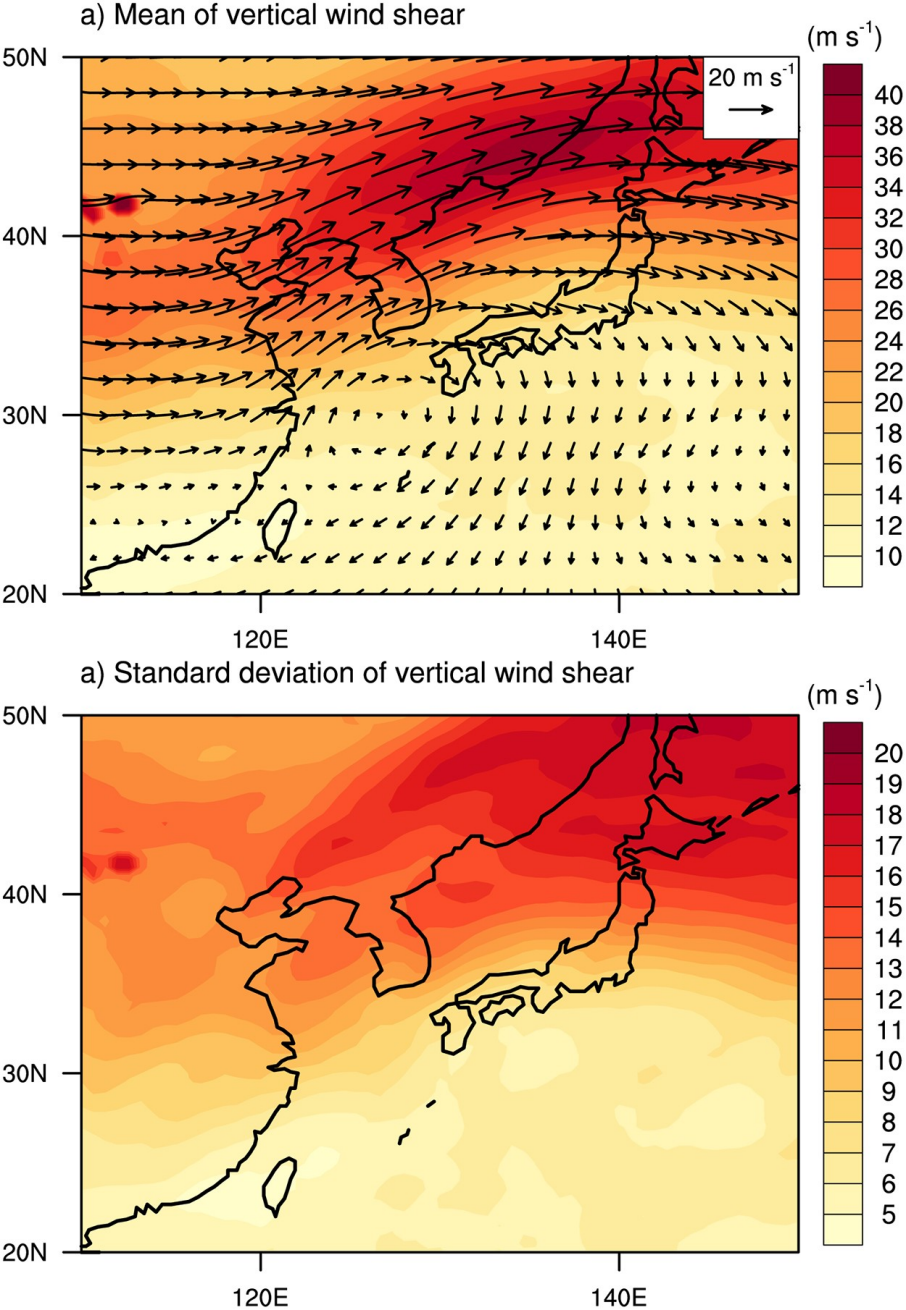
The (a) mean and (b) standard deviation of vertical wind shear over the East Asia region during the landfall period.

Prior to analyzing the variation of wind- and rainfall areas, composite structures of horizontal winds and rainfall for the entire 283 samples during the landfall periods were examined. The composites were obtained in a rotated coordinate system in which the directions of TC motions are oriented due north (i.e., parallel to the y-axis of a rectangular domain). The mean 10-m wind field depicts well-organized cyclonic circulation with distinct asymmetry in the left and right sides of the TC motion (Fig 3a). This wavenumber-1 asymmetry is a well-known feature of moving TCs [46, 48, 51, 55]. The rainfall area lies mainly in the front side of the TC (Fig 3b) and is attributed to the asymmetric wind structure inducing surface convergence and upper-tropospheric divergence to promote vigorous convection and heavy rainfall there (Fig 3c and 3d). Note that most of TCs present their maximum wind and rainfall in the right and front side, respectively (Table 1). In addition, southwesterlies are dominant in the upper troposphere (Fig 3d), implying that TCs were strongly affected by the westerly jet prevailing in the mid-latitudes. Fig 4 shows the environmental vertical wind shear over East Asia during the landfall period. It is noteworthy that the vertical wind shear over South Korea is very strong (mean value > 20 m s^-1^; Fig 4a) and shows large variation between samples (standard deviation > 10 m s^-1^; Fig 4b). This implies that TCs landfalling in South Korea can be significantly affected by baroclinic environments (i.e., environmental vertical wind shear); this will be discussed in the next section. The direction of vertical wind shear (Fig 4a) is similar to the direction of TC motions, since the moving direction of TCs landfalling in South Korea is generally north-eastward (Fig 1).

**Table 1:** The occurrence numbers of maximum wind and rainfall in the center region, and four quadrants (front, right, rear, and left) out of the center region. The center region is defined as the region within 100 km from the TC center. The direction of four quadrants are based on the direction of TC motion.

	Center	Front	Right	Rear	Left
Maximum wind	26	39	172	29	17
Maximum precipitation	53	114	55	41	20

**Fig 5:**
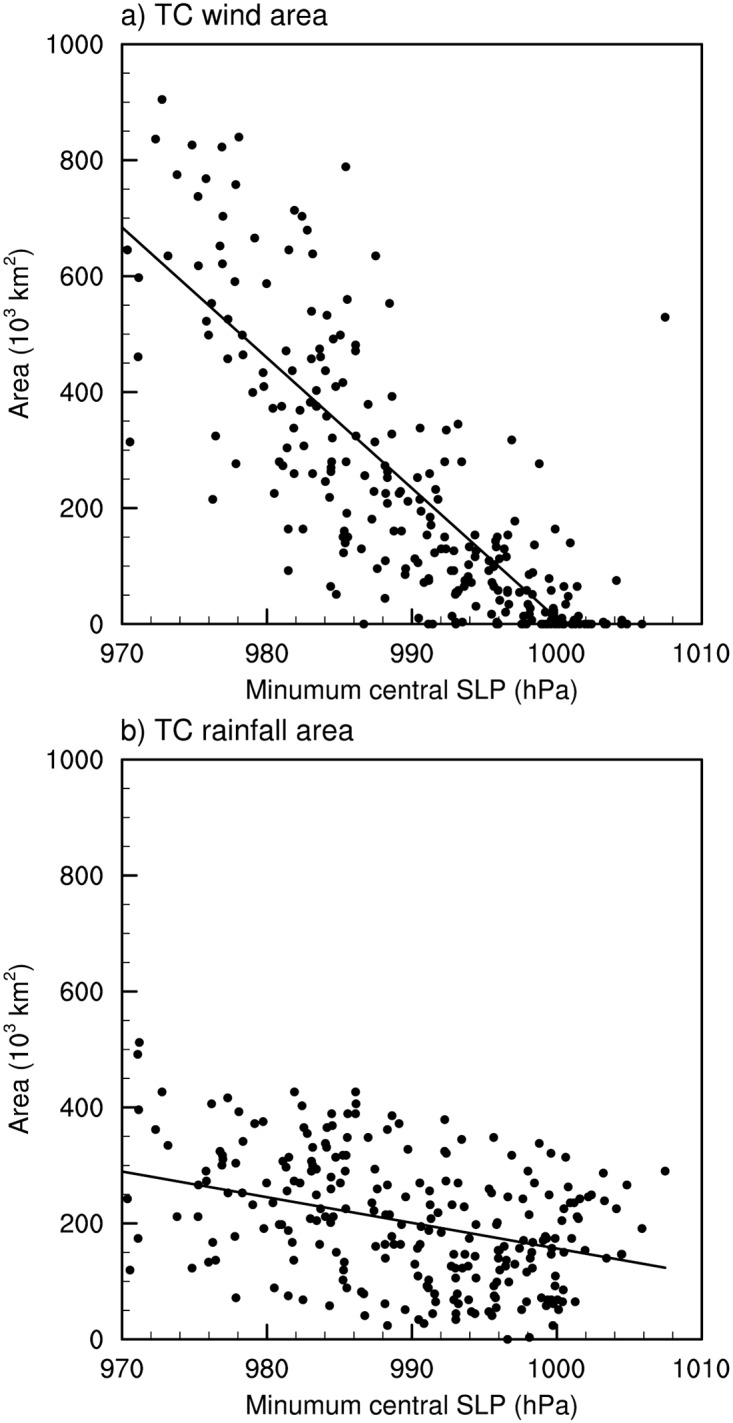
Distributions of the (a) wind area, and (b) rainfall area according to the minimum central SLP. The solid line in each plot denotes the linear regression line.

Fig 5 displays the relationship of the areal extent to the TC intensity. Both the wind- and rainfall areas are negatively correlated with the minimum central SLP with correlation coefficients of −0.86 and −0.45, respectively, which are significant at the 95% confidence level. This result indicates that stronger TCs are generally associated with larger areal extents. Many studies, however, suggested insignificant relationship between the intensity and the areal extents of TCs [4, 5, 9, 10]. The difference between this and previous studies are not due to the different definition of the areal extent (i.e., radial and areal size), since the wind area defined in this study shows positive relationship (*r* = 0.73; significant at the 95% confidence level) with the wind radii of 34-kt in the best track data. The wind radii of 34-kt and the minimum central SLP obtained from the best track data also showed significant negative correlation coefficient (*r* = −0.69; significant at the 95% confidence level) over South Korea. In addition, Chavas et al. [11] presented that the outer sizes of TCs tend to increase with TC intensities up to a certain range (about maximum wind speed of 40 m s^-1^ in their study). Since TCs are generally weaker in the landfall period than in the mature stage, the negative relationship between the areal extent and the minimum central SLP may exist only in the vicinity of South Korea.

### Variation of wind- and rainfall areas by vertical wind shear

**Fig 6:**
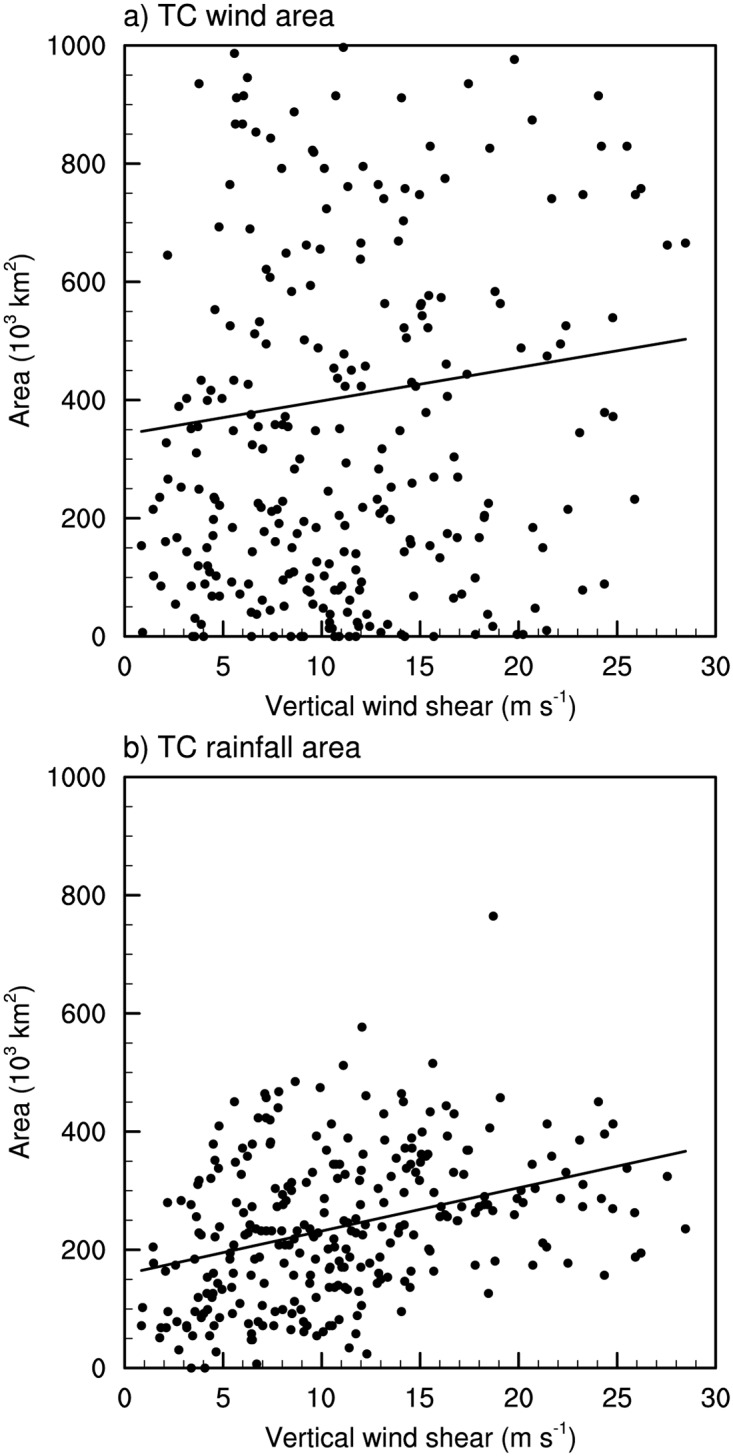
Distributions of the (a) wind area, and (b) rainfall area according to the vertical wind shear. The solid line in each plot denotes the linear regression line.

As seen in Fig 4, strong environmental vertical wind shear with large variation appears over South Korea during the landfall period. To examine the influence of the vertical wind shear on the variation of the wind- and rainfall areas, the vertical wind shear within a TC is defined as the difference between the mean wind vectors of the 250- and 850-hPa levels over an annular region of 300–800 km around the TC center. Fig 6 shows the distribution of the wind- and rainfall areas according to the vertical wind shear within TCs. No significant relationship is found between the wind area and vertical wind shear (*r* = 0.07); however, the rainfall area significantly increases with vertical wind shear (*r* = 0.37; significant at the 95% confidence level). The mean and standard deviation of the vertical wind shear affecting TCs are 11.03 m s^-1^ and 5.95 m s^-1^, respectively. To examine the detailed effects of the vertical wind shear, TCs in strong shear cases (i.e., SS cases) are compared against those in weak shear cases (i.e., WS cases). The SS and WS cases are defined in terms of the magnitude of the vertical wind shear; the wind shear in the SS cases exceeds the all-case mean value plus half of the standard deviation (14.01 m s^-1^) and the WS cases are of vertical wind shear smaller than the all-case mean value minus half of the standard deviation (8.05 m s^-1^). Applying the conditions, 82 (102) SS (WS) cases are identified.

**Fig 7:**
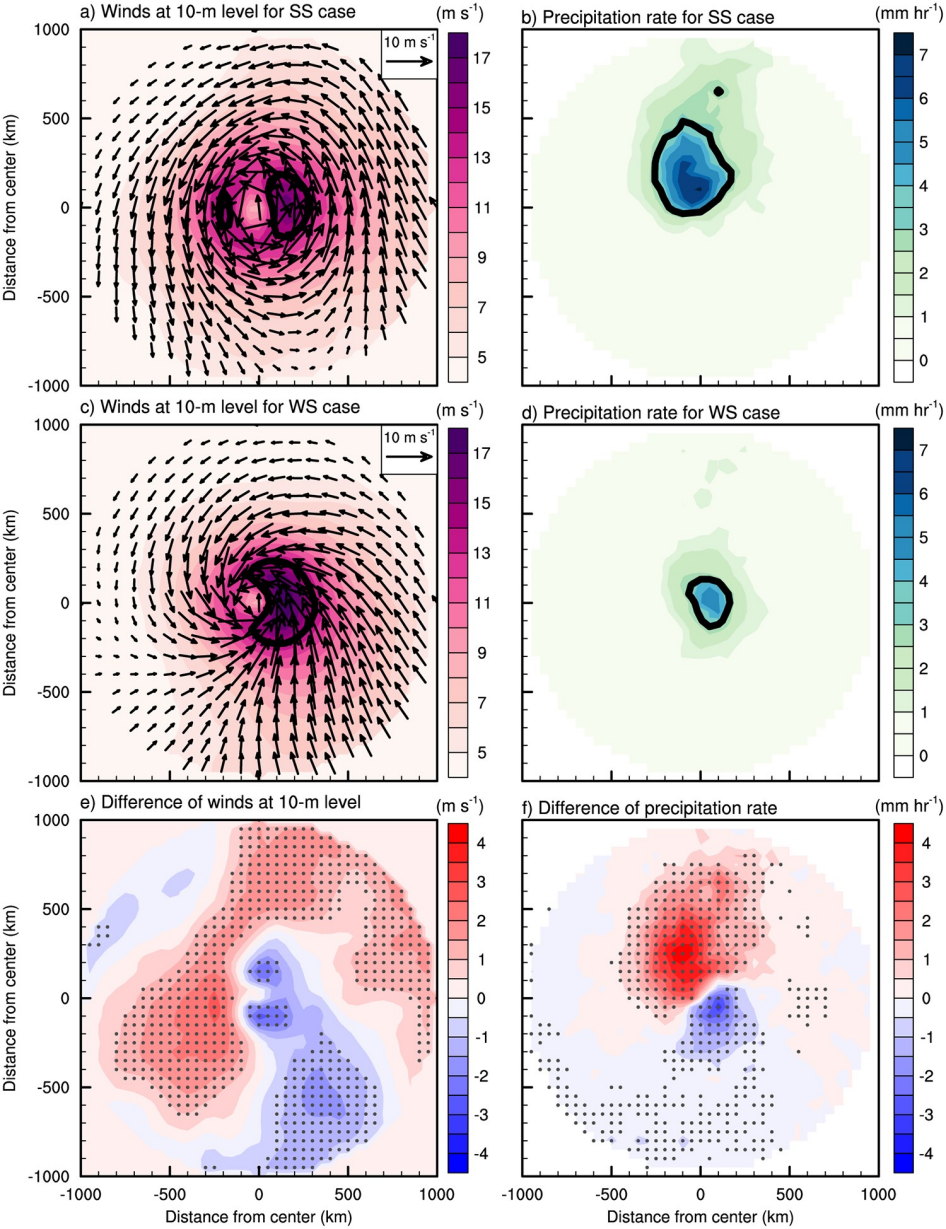
The averaged horizontal TC structure of wind at 10-m level (arrows, unit: m s^-1^) with its speed (shaded, unit: m s^-1^), and precipitation rate (unit: mm hr^-1^) for the (a, b) strong shear (SS) case, (c, d) weak shear (WS) case, and (e, f) their differences (SS minus WS) during the landfall period. The directions of x- and y- axis are right and front to TC motion, respectively. The gray dots indicate the statistically significant differences at the 95% confidence level evaluated with Student’s *t*-test.

The TC wind and rainfall structures vary distinctly between the SS and WS cases. Fig 7 displays the composite structures of surface wind at 10-m level and rainfall for the two cases, as well as their differences. Both cases show the cores of maximum wind in the right side of the TC moving direction (Fig 7a and 7c) with the SS cases showing weaker (stronger) winds in the right (left) side compared to the WS cases (Fig 7e). Thus, there is no significant difference in the wind area between the two cases (Table 2). On the other hand, the rainfall area in the SS cases shows a notable asymmetric structure, while WS cases show nearly symmetric rainfall (Fig 7b and 7d). The rainfall area in the SS cases is significantly enhanced (slightly reduced) in front (behind) of TCs compared to the WS cases (Fig 7f). Consequently, the rainfall area is significantly larger (about 1.5 times on average) in the SS cases than in the WS cases (Table 2). The TC intensities are similar in the two cases.

**Table 2:** The average vertical wind shear, wind area, rainfall area, and minimum sea level pressure of TCs for the strong and weak shear cases. Statistically significant differences between the two cases for vertical wind shear and rainfall area appear at the 95% confidence level evaluated with Student’s *t*-test.

Case	Sample number	Vertical wind shear (m s^-1^)	Wind area (10^3^ km^2^)	Rainfall area (10^3^ km^2^)	Min SLP (hPa)
Strong shear	82	18.6	324.6	276.4	976.7
Weak shear	102	5.2	304.9	176.4	977.3

**Fig 8:**
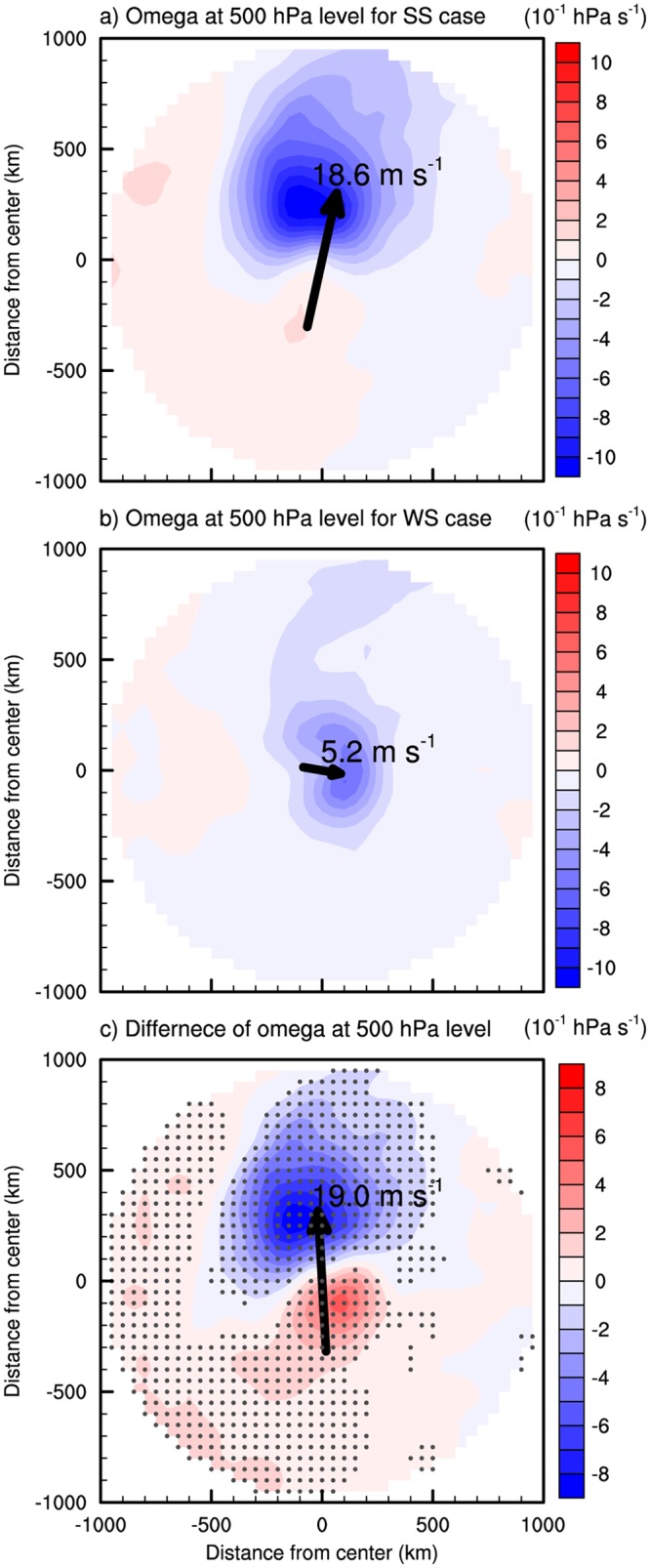
The vertical velocity (shaded, unit: 10^−1^ hPa s^-1^) at 500 hPa level and the vertical wind shear vector (arrow, unit: m s^-1^) in the (a) SS case, (b) WS case, and (c) their differences (SS minus WS) during the landfall periods. The directions of x- and y- axis are right and front to TC motion, respectively. The gray dots indicate the statistically significant differences at the 95% confidence level evaluated with Student’s *t*-test.

The SS and WS cases also show significantly different dynamic structures. The SS cases show anomalous rising and sinking motions in the front and rear side of the TC, respectively, compared to the WS cases (Fig 8). It is notable that the difference of the vertical velocity (Fig 8c) matches closely the difference of rainfall (Fig 7e). This dipole pattern of vertical velocity difference indicates the occurrence of asymmetric convection within TCs. A number of studies showed that the asymmetric convection in TCs can be induced by vertical tilting by wind shear [47–49, 56–58]; a TC experiencing strong environmental vertical wind shear can induce rising and sinking motions in the downshear-left and upshear-right side, respectively [50, 51, 58, 59]. Note that the average vertical wind shear vectors (black arrows in Fig 8) and associated vertical motions in TCs found in this study are consistent with the results reported in earlier studies. In both cases, strong rising motions appear in the downshear-left side while there are almost no rising motions in the upshear-right side. The SS cases shows stronger vertical wind shear (18.6 m s^−1^) than the WS case (5.2 m s^−1^) (Table 2; Fig 8a and 8b). Thus, the difference of shear vector (19.0 m s^−1^) between the two types of TCs can induce the relative rising and sinking motions at the front and rear side of TC, respectively (Fig 8c).

**Fig 9:**
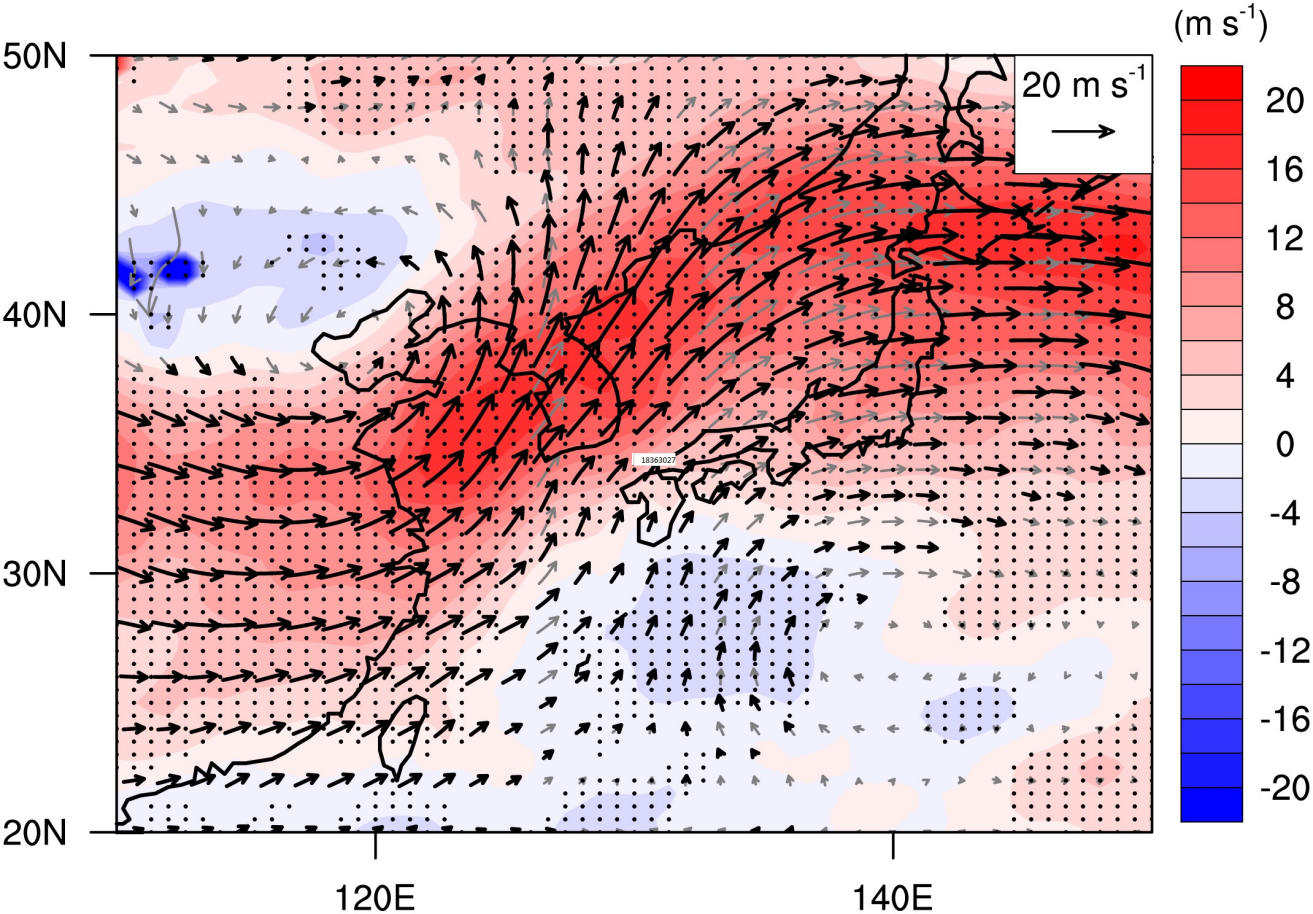
The difference composite (SS minus WS) of vertical wind shear (arrows, unit: m s^-1^) and its magnitude (shaded, unit: m s^-1^) between 850 and 250 hPa level. The thick black arrows and gray dots indicate the statistically significant differences at the 95% confidence level evaluated with Student’s *t*-test.

The difference of the environmental condition in the mid-latitude East Asia between the SS and WS cases supports that the difference of the vertical wind shear within TCs (Fig 8) are induced by the large-scale circulation. For the SS cases, significantly stronger vertical wind shear appears in the mid-latitude East Asia compared to the WS cases (Fig 9). This strong vertical wind shear is associated with the upper-level trough located in the northeastern China near Beijing. The upper-level trough plays a key role in rapid intensification of TCs [60–62] as well as the changes in the TC structure such as extratropical transition (ET) [62, 63]. In particular, Hanley et al. [64] showed that the vertical circulation of a TC can be significantly changed while horizontal wind is not intensified when the TC is affected by the upper-level trough accompanied by strong vertical wind shear (larger than 10 m s^-1^) as also found in this study. In addition, the ET process can be closely related to the findings in this study. In the mid-latitudes, a number of TCs show significant changes such as frontogenesis, vertical tiling, abrupt intensification of wind and rainfall, during the ET process [65–67]. Indeed, most of the TCs (about 90%) reaching the mid-latitude (> 30°N) region in the WNP show ET processes during the analysis period in this study (1998–2013). The vertical tilting and asymmetric vertical motion are also frequently observed in the ET process [68, 69].

**Fig 10:**
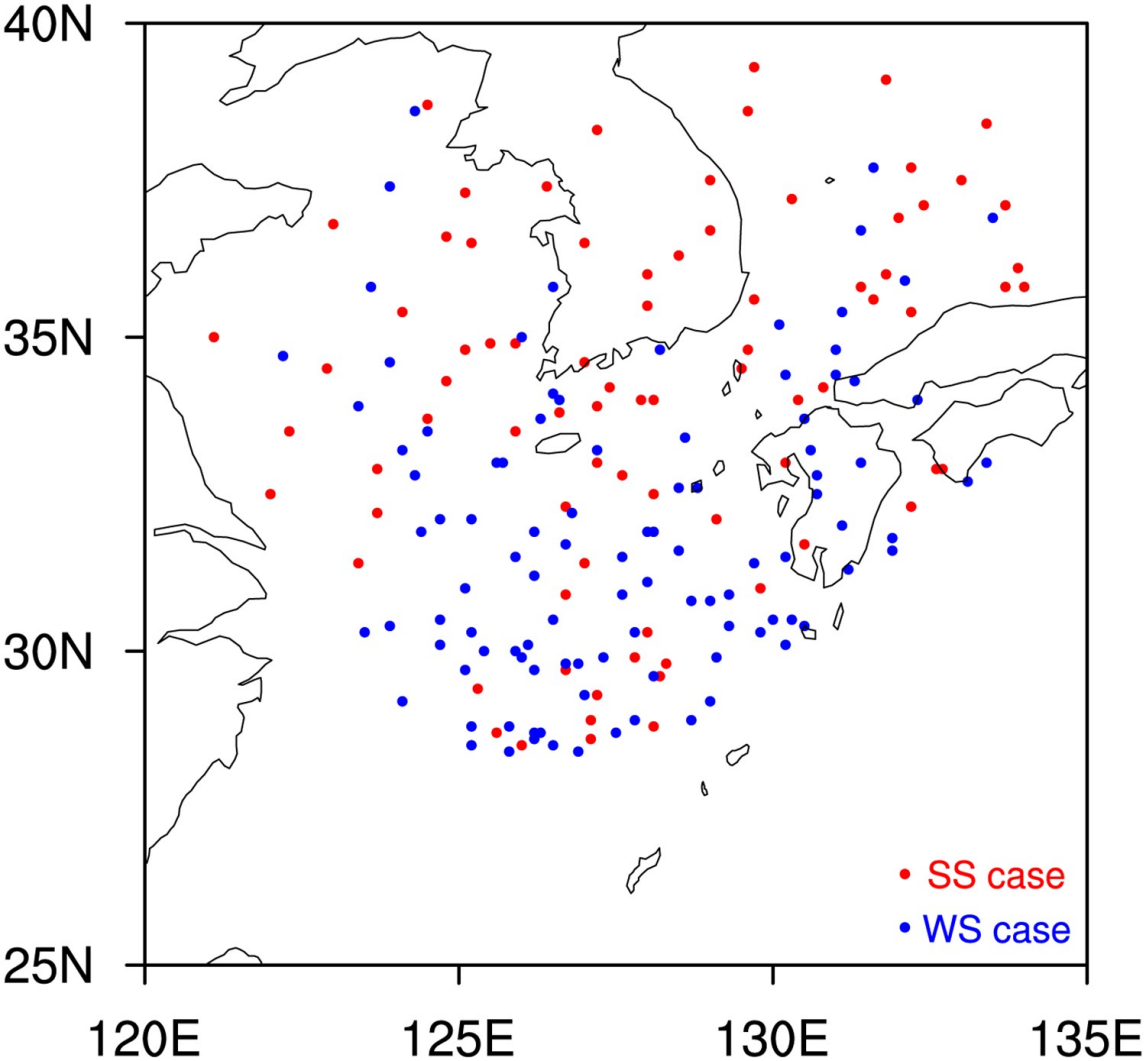
The distribution of TC center for the SS (red) and WS (blue) cases.

Fig 10 presents the spatial distribution of TC centers for the SS and WS cases. The mean latitude of the TC centers in the SS cases (34.1°N) is significantly higher (95% confidence level) than that for the WS cases (31.9°N) consistently with the distribution of environmental vertical wind shear (i.e. stronger shear in higher latitude; Fig 4). There is no significant difference of the mean longitude of the TC center between the SS and WS cases (128.1°E and 127.7°E, respectively). In the landfall period, the wind area of TCs can be reduced due to the increasing surface friction while the rainfall area of TCs can be enhanced by topography [70–73]. Such effects of topography may contribute to the variation of rainfall areas in the SS and WS cases. However, TCs are not confined within land areas for both the SS and WS cases (Fig 10). Thus, the overall results in this study would remain valid even with topographic effects.

## Summary and discussion

The wind- and rainfall areas of TCs making landfall in South Korea during 1998–2013 are investigated using the MERRA-2 reanalysis and TRMM-3B42 precipitation data. The wind- and rainfall areas are defined as the regions where wind speeds and precipitation rates exceed their threshold values, 14 m s^−1^ and 3.33 mm hr^−1^, respectively. TCs making landfall in South Korea present strong winds and heavy rainfall in the right and front side of the TC moving direction, respectively. Strong vertical wind shear appears over South Korea during TC landfalls as well. This study focuses on the effects of the vertical wind shear on the wind- and rainfall areas of landfalling TCs. It has been found that the vertical wind shear appearing over South Korea during TC landfalls is significantly related to the increase of TC rainfall area by inducing asymmetric convection characterized by rising and sinking motion at down-shear left and up-shear right side, respectively. On the other hand, the TC wind areas are not affected by the vertical wind shear.

This study suggests that it is necessary to consider the asymmetry of wind- and rainfall areas in preparing to minimize the damages by TC landfalls. In addition, various environmental conditions need to be considered in developing an accurate forecasting system for the areas primarily affected by strong winds or heavy rainfall. According to our results, the environmental vertical wind shear over South Korea is a crucial factor in shaping the TC rainfall area in South Korea. However, the wind- and rainfall areas can be affected not only by the vertical wind shear but also by various environmental conditions which varies substantially according to regions [e.g., 11, 19, 20]. Thus, further studies on other regions are necessary to improve our understanding of the areas affected by TC winds and rainfall. Numerical model experiments will also be necessary to study the detail mechanisms for the variation of TC wind- and rainfall areas.
